# Development and Validation of the Idiopathic Multicentric Castleman Disease Symptom Burden Scale (ISBUS): Protocol for an International Multistage Mixed Methods Study

**DOI:** 10.2196/96022

**Published:** 2026-08-11

**Authors:** Philip A Powell, Anju Keetharuth, Sé Maria Frances, Jill Carlton, Kelly Makarounas-Kirchmann, Antonio Adolfo Guerra Soares Brandão, Karthik Ramasamy, Francis Shupo, Kelley C Dacus, Corey Casper, David C Fajgenbaum, Yeow Goh, Frits van Rhee, Pier Luigi Zinzani, Éric Oksenhendler, Lu Zhang, Sudipto Mukherjee

**Affiliations:** 1 School of Medicine and Population Health University of Sheffield Sheffield, England United Kingdom; 2 KMC Health Care Melbourne Australia; 3 Hospital BP – A Beneficência Portuguesa de São Paulo São Paulo Brazil; 4 Nuffield Department of Orthopaedics, Rheumatology and Musculoskeletal Sciences University of Oxford Oxford, England United Kingdom; 5 Oxford University Hospitals NHS Trust Oxford, England United Kingdom; 6 Recordati Rare Diseases Hemel Hempstead, England United Kingdom; 7 Recordati Rare Diseases Inc. Bridgewater, NJ United States; 8 Banner Health Phoenix, AZ United States; 9 College of Medicine University of Arizona Phoenix, AZ United States; 10 University of Pennsylvania Philadelphia, PA United States; 11 Singapore General Hospital Singapore, SG.01 Singapore; 12 University of Arkansas for Medical Sciences Little Rock, AR United States; 13 Istituto di Ematologia “Seràgnoli” IRCCS Azienda Ospedaliero-Universitaria di Bologna Bologna Italy; 14 Dipartimento di Scienze Mediche e Chirurgiche University of Bologna Bologna, Emilia-Romagna Italy; 15 Université de Paris Paris, Île-de-France France; 16 Department of Clinical Immunology Hôpital Saint-Louis Paris, Île-de-France France; 17 Department of Hematology Peking Union Medical College Hospital Chinese Academy of Medical Sciences & Peking Union Medical College Beijing, Beijing China; 18 Department of Hematology and Medical Oncology Cleveland Clinic Cleveland, OH United States

**Keywords:** clinical outcome assessment, COA, idiopathic multicentric Castleman disease, iMCD, meaningful change, minimal clinically important difference, patient-centered outcome, patient-reported outcome measure, PROM, psychometrics, qualitative research, symptom burden

## Abstract

**Background:**

Idiopathic multicentric Castleman disease (iMCD) is a rare lymphoproliferative disorder that is associated with a broad range of symptoms, including constitutional, gastrointestinal, neuropsychiatric, dermatologic, respiratory, and hematologic or lymphoreticular problems. These broad symptoms can impact the daily lives of people living with iMCD, creating a high symptom burden. Despite this, no robust, disease-specific patient-reported outcome measure (PROM) for subjective iMCD symptom burden exists. This limits accurate symptom monitoring, impacts sensitive end point selection in clinical trials, and represents a regulatory gap in patient-centered evidence generation when evaluating iMCD treatments.

**Objective:**

This protocol describes the international multistakeholder Idiopathic Multicentric Castleman Disease Symptom Burden Scale (ISBUS) project. The primary aim of this project is to develop and preliminarily evaluate the measurement properties of a novel PROM for capturing symptom burden (ie, perceived symptom frequency and impact on daily life) in people living with iMCD. The central objective is to produce a valid and robust PROM that can be used to assess iMCD symptom burden in research, clinical trials, and symptom monitoring in clinical practice.

**Methods:**

The project has a mixed methods design, split into 4 sequential stages, with collaborative advisory input throughout. Stage 1 uses existing data and consultation with expert clinical and patient advisors to generate draft PROM content. Stage 2 uses qualitative cognitive debriefing interviews (n=10) to evaluate the content validity of the draft PROM content and enable meaningful revisions. Stage 3 involves a cross-sectional quantitative survey design in people living with iMCD (n≥50), allowing for psychometric analyses of structural validity (using classical test theory and/or Rasch methodology), construct validity (known-group validity and correlational methods), and internal consistency reliability (Cronbach α). Stage 4 uses a mixed methods design, including longitudinal quantitative survey evidence (n≥20) and qualitative interviews (n=10), triangulated to estimate preliminary meaningful change estimates for the new PROM. The project is international in scope, with primary data collection in 6 countries (ie, Australia, Brazil, Canada, New Zealand, the United Kingdom, and the United States).

**Results:**

Funding for ISBUS began in June 2023 and is ongoing. As of March 2026, stage 3 had been completed, with 51 people living with iMCD completing the survey, and stage 4 was ongoing, with 32 follow-up surveys completed. Analyses for stages 1-3 were completed and are expected to be published in 2026, followed by stage 4 findings in 2027.

**Conclusions:**

The aim of the ISBUS project is to produce a novel symptom burden PROM codeveloped with patients with iMCD to measure what matters to patients with iMCD. It is anticipated that the new PROM will be used in iMCD research, as a secondary end point in trials, and as a clinical tool to monitor symptom burden in the management of iMCD.

**Trial Registration:**

ClinicalTrials.gov NCT05995834; https://clinicaltrials.gov/study/NCT05995834

**International Registered Report Identifier (IRRID):**

DERR1-10.2196/96022

## Introduction

Castleman disease encompasses a heterogeneous group of cytokine-driven lymphoproliferative disorders that share a common lymph node histopathologic appearance. Classification is based on the number of lymph node stations involved: unicentric Castleman disease involves a single lymph node station, whereas multicentric Castleman disease (MCD) involves multifocal lymphadenopathy. MCD is further classified based on etiology, including MCD caused by human herpesvirus 8 (HHV-8) infection; idiopathic multicentric Castleman disease (iMCD) associated with polyneuropathy, organomegaly, endocrinopathy, monoclonal plasma cell disorder, and skin changes (POEMS) and driven by a monoclonal plasma cell population; and iMCD, where the etiology is unknown [1]. Further diagnostic differentiation of HHV-8–negative and HIV-negative iMCD into subtypes is based on clinicopathologic presentation, including iMCD associated with thrombocytopenia, anasarca, fever, reticulin fibrosis, and organomegaly; iMCD associated with idiopathic plasmacytic lymphadenopathy; and the remainder of iMCD cases classified as not otherwise specified [2-4].

iMCD is a rare condition with an estimated prevalence of 6.9 to 9.7 per million people [5]. This disease is characterized by a chronic hyperinflammatory state with a clinical trajectory notable for high and fluctuating symptom burden and multiple morbidities resulting from organ dysfunction [1,6,7]. Currently, no diagnostic biomarker for iMCD exists, and diagnostic criteria were established only recently, in 2017 [1]. While clinical presentations vary, iMCD can be associated with a high symptom burden, including constitutional, gastrointestinal, neuropsychiatric, dermatologic, respiratory, and hematologic or lymphoreticular problems [8]. Both disease-based symptoms and treatment side effects can impact the health-related quality of life (HRQoL) of people living with iMCD [8,9].

There is no cure for iMCD, and given the potentially significant symptom burden, the mainstay of clinical management remains symptom alleviation and mitigation of organ complications. Moreover, for several symptoms, there are no specific laboratory or radiologic correlates; therefore, those symptoms are not captured. Additionally, there is currently one approved medication for iMCD [10], and guidelines exist on the therapeutic management of patients with iMCD [11,12]. Despite the evidence supporting the efficacy [10] and effectiveness [13] of this medication for the treatment of iMCD, a substantial proportion of patients with iMCD are not provided with any therapeutic modality, and many patients do not receive recommended first-line therapy [5,14]. Taken together, accurately and reliably assessing symptom presentation and its impact on patients’ lives is critically important.

However, research to date on patient-reported symptom burden has been limited [8,9]. To the authors’ knowledge, only 1 validated patient-reported outcome measure (PROM) for MCD exists, the MCD-Symptom Scale (MCD-SS), which measures symptom severity [15,16]. However, there are several limitations of the MCD-SS. First, although the MCD-SS followed the US Food and Drug Administration (FDA) guidelines for PROM development [17], there is insufficient information regarding the methodology, and it has not been subject to independent peer review, with supporting evidence presented only at conferences. Second, it was derived based on the cumulative input of 12 patients (from 2 US centers) and 7 clinicians, raising concerns regarding the generalizability of the results, particularly to specific condition subtypes such as iMCD. Third, this scale does not explicitly capture the perceived impact of symptoms on patients’ daily lives, capturing symptom severity only, which is not delineated from symptom presence. Lastly, and most importantly, the scale focuses on MCD and not necessarily iMCD. The MCD-SS predated the development and publication of the international iMCD diagnostic criteria; therefore, it is unlikely that the patients involved in the development of the MCD-SS included appropriately diagnosed patients with iMCD [1]. Apart from its use in a phase 2 trial of siltuximab for patients with MCD [10,16], it has been used sparingly in peer-reviewed clinical research, with only 1 instance of its application in a cross-sectional observational study in China [18].

Alternatively, existing generic measures of symptom burden or HRQoL are typically not adapted for use or sufficiently sensitive to capture aspects of burden that matter to people living with rare diseases, such as iMCD [19]. Therefore, a clear gap exists for an appropriate, peer-reviewed, and quality-assured measure of symptom burden that measures what matters to people living with the condition and performs well on psychometric indices of validity and reliability. Regulatory bodies, such as the FDA, require patient-centered evidence to support treatment evaluation and labeling claims and have produced guidelines to assist with the development of PROMs for this purpose [17]. This guidance should be balanced with additional practical considerations for outcome measures in rare diseases [19,20]. Accordingly, the Idiopathic Multicentric Castleman Disease Symptom Burden Scale (ISBUS) will involve a multistage, mixed methods process and the key involvement of patients throughout, as both informed participants and peer-to-peer collaborative advisors [21]. A previous bespoke iMCD symptom burden survey conducted in 2023 by Mukherjee et al [8] forms the starting point for further development work. Additional research is needed to produce a standardized and transferable PROM, including cognitive interviewing, psychometric analyses, and advisory input from patients and clinicians [17].

The ISBUS study [22] is an international multistakeholder project conceived through collaboration among industry, clinicians, and patient representatives to design an outcome measure to meet the unmet need to quantify symptom burden in iMCD. The primary aim of this project is to develop and preliminarily evaluate the measurement properties of a novel PROM for capturing symptom burden in people living with iMCD. Symptom burden is typically conceptualized as a multidimensional concept encompassing both the prevalence of multiple symptoms (via their frequency and/or severity) and the perceived impact of these symptoms on the individual experiencing them [23]. This concept has been applied in multiple disease areas. For example, the Memorial Symptom Assessment Scale–Heart Failure asks patients about the frequency, severity, and distress associated with 32 possible symptoms linked to heart failure [24]. Symptom burden is calculated by taking the mean of the 3 responses. Similarly, in cancer, the Patient-Reported Outcomes–Common Terminology Criteria for Adverse Events features items with 1 to 3 ratings of frequency, severity, and/or interference with daily activities, which can be combined into a composite burden score for symptoms experienced during clinical trials [25,26].

**Figure 1:**
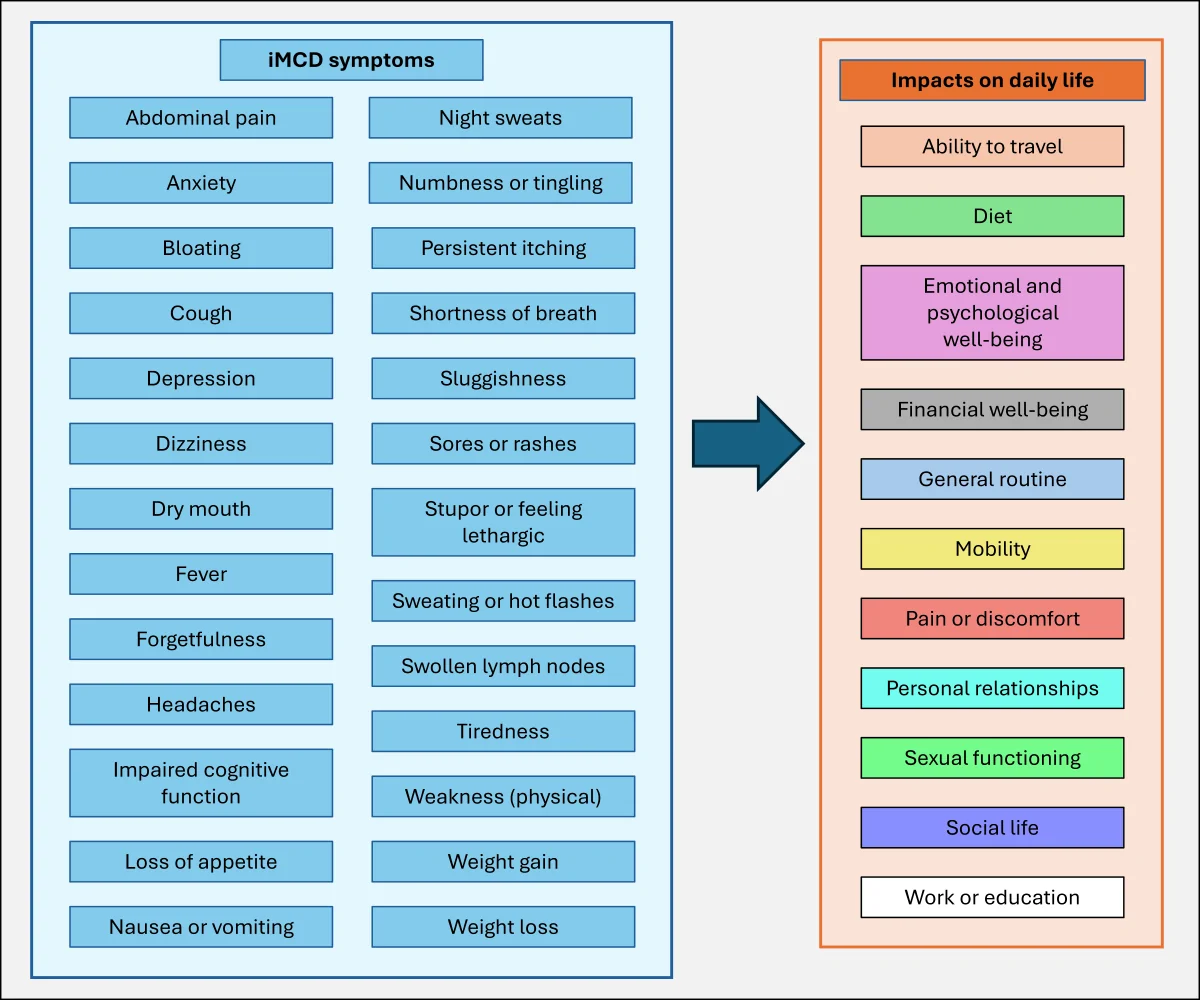
Draft conceptual model of symptom burden in idiopathic multicentric Castleman disease (iMCD).

**Figure 2:**
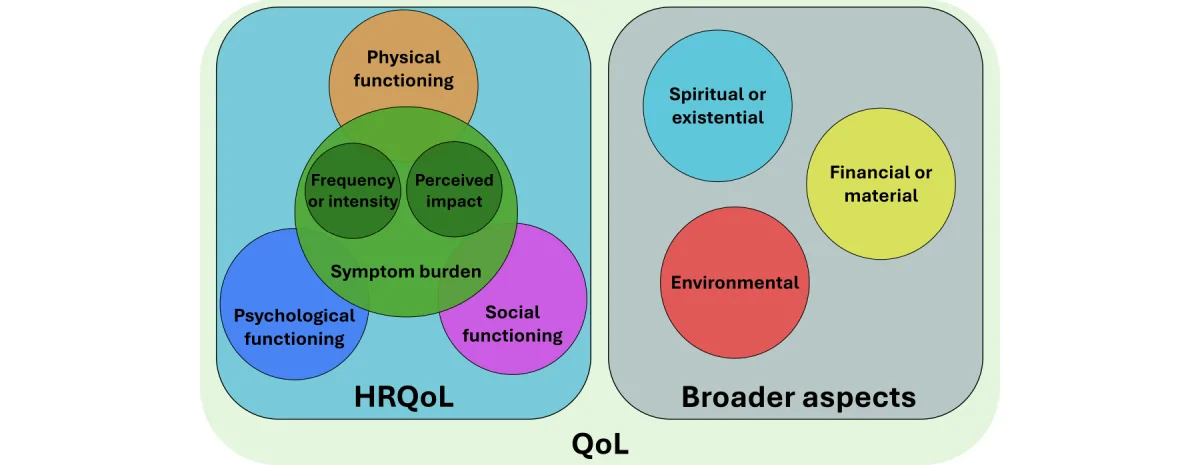
Conceptual model of symptom burden. Symptom burden is conceptualized as a combination of symptom frequency or symptom intensity and perceived impact on the patient. Symptom burden is a determinant of health-related quality of life (HRQoL) via its relationship with physical, psychological, and social functioning, which may be bidirectional. HRQoL is a subcomponent of quality of life (QoL), which includes broader aspects, such as spiritual, financial, and environmental well-being.

In our previous research on iMCD, we identified 26 unique symptoms from a patient survey that impacted at least 11 domains of patients’ daily lives, including physical, mental, and social aspects [8]. These findings informed a condition-specific conceptual model of iMCD symptom burden that guides the design and development of ISBUS (Figure 1). Separately, at a higher level, symptom burden can be conceptualized as a determinant of patients’ HRQoL via its relationship with core domains, such as physical, psychological, and social functioning, recognized as integral to common HRQoL models [27]. Figure 2 presents a more generic conceptual model of the perceived relationship between symptom burden and HRQoL adopted for this study, which helps situate the construct within a wider quality-of-life framework.

The intended primary context of use of the novel measure will be in iMCD research and as a secondary end point in clinical trials, but further confirmatory studies may assess the utility of the measure in clinical settings and its role in disease management. The project aim will be achieved across 4 research stages, each with its own unique objectives:

- Item generation: based on existing patient data and advisory input (from both patients and clinicians), derive a comprehensive long list of candidate items (and the draft structure of the scale, including response options) for the ISBUS.
- Item testing and refinement: based on cognitive interviews with people living with iMCD, assess and summarize the content validity of the draft items. Revise the draft PROM based on this evidence collaboratively with advisory input.
- Final item selection: administer the draft PROM alongside complementary questions and conduct appropriate psychometric analyses. Considering all the evidence collected, agree with advisors on the final symptom burden scale.
- Preliminary measures of change: using a readministered scale (quantitative evidence) and interviews with participants (qualitative evidence), estimate a minimal clinically important difference, as agreed with advisors.

## Methods

### Study Design

**Figure 3:**
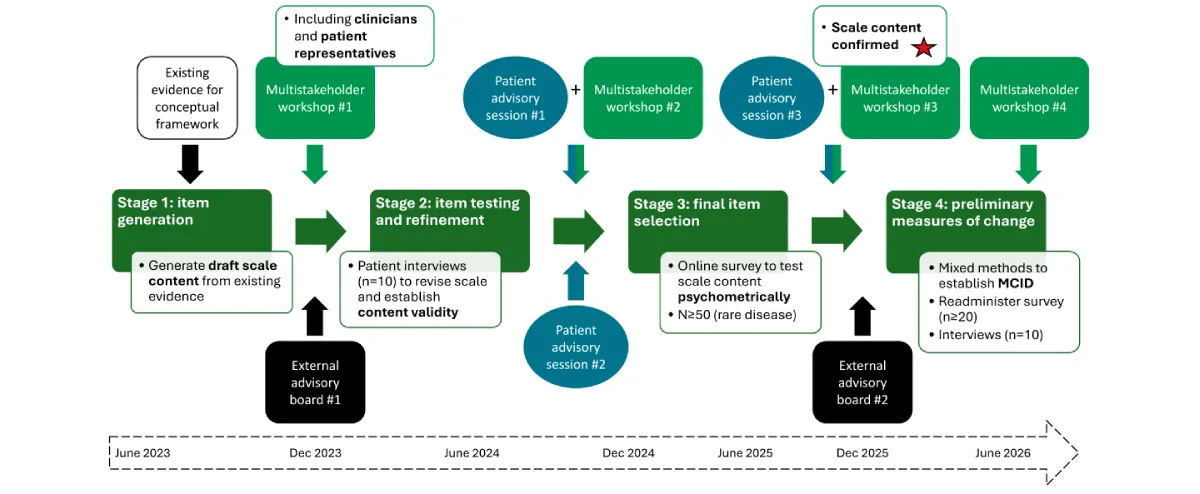
Four-stage Idiopathic Multicentric Castleman Disease Symptom Burden Scale (ISBUS) project design. The ISBUS project proceeds through 3 main stages of patient-reported outcome measure development (stages 1-3) and a fourth stage exploring preliminary, observational estimates of meaningful change. Collaborative patient and expert advice are embedded throughout the project through dedicated meetings and workshops at key stages. Work on the project began in June 2023 and is expected to conclude by August 2026. Dec: December; MCID: minimal clinically important difference.

This project has a mixed methods design, split into 4 sequential stages, with collaborative advisory input throughout (Figure 3). Stage 1 uses existing data and consultation with expert clinical and patient advisors to generate draft PROM content. Stage 2 uses a qualitative design to evaluate the content validity of the draft PROM and facilitate revisions. Stage 3 involves a cross-sectional quantitative survey design in people living with iMCD. Stage 4 uses a mixed methods design, including longitudinal quantitative evidence and qualitative interviews, to estimate preliminary meaningful change thresholds for the new scale. The project is international in scope, with primary data collection in 6 countries (ie, Australia, Brazil, Canada, New Zealand, the United Kingdom, and the United States), as described below.

### Project Governance and Advisory Groups

Consistent with best practice guidance in scale development [19,21,28], ISBUS embeds engagement with patients and clinical experts throughout the project. Specifically, the project involves 2 internal core advisory groups. First, the Patient Advisory Group (PAG) consists of 4 to 6 people with lived experience of iMCD. These collaborators take part in dedicated and exclusive patient advisory sessions with researchers and have representative input into multistakeholder meetings. Second, the Multistakeholder Advisory Group (MAG) includes 2 to 3 clinical experts in iMCD and 1 to 3 patient representatives from the PAG. The MAG acts as the primary internal decision-making body throughout the project, ratifying decisions at each stage of scale development. The project also features an External Advisory Board, including 5 to 10 clinical experts in Castleman disease, with some intentional overlap with the MAG (ie, 3 clinicians who are also ISBUS investigators or coinvestigators).

For the PAG and MAG roles, terms of reference and role descriptions are provided to collaborators outlining expected duties and contributions throughout the project. Collaborating patients in most countries, except Brazil, where ethics approval prohibited the payment of patients, are reimbursed for their time and contributions in line with UK National Institute for Health and Care Research guidance at the time [29]. Collaborators received compensation equivalent to 25 units of local currency per hour (eg, GBP £25 in the United Kingdom, US $25 in the United States). The role of each advisory group is described in Table 1.

**Table 1:** Patient and expert clinician involvement in developing the ISBUS^a^.

Stage and activity		Purpose	People involved, n
Stage 1: item generation			
	MAG^b^ 1	Reach consensus on the draft scale content and which items will be tested for inclusion in the scale	Clinician collaborators (2-3) Patient collaborator representatives (1-3)
	EAB^c^ 1	Expert feedback on study design, patient screening criteria, and decisions on draft scale content (MAG 1)	International experts in Castleman disease (5-10)
Stage 2: item testing and refinement			
	Cognitive interviews	Test the content validity of draft PROM^d^ (ie, comprehensibility, relevance, and comprehensiveness)	Patients as research participants (10)
	PAG^e^ 1	Discuss and help interpret cognitive interview results and coagree decisions on scale refinements	Patient collaborators (4-6)
	MAG 2	Discuss cognitive interview results and patient input (PAG 1); finalize content for stage 3	Clinician collaborators (2-3) Patient collaborator representatives (1-3)
Stage 3: final item selection			
	PAG 2	Discuss and agree content of proposed online survey, including bespoke questions on iMCD^f^ and treatment	Patient collaborators (4-6) Other stakeholders (as relevant)
	Online survey (time 1)	Gather cross-sectional survey data for psychometric analysis (time 1)	Patients as research participants (>50)
	PAG 3	Discuss and help interpret survey results and coagree decisions for finalized scale	Patient collaborators (4-6)
	EAB 2	Discuss survey results. Advise on decision for the final PROM content	International experts in Castleman disease (5-10)
	MAG 3	Discuss and finalize decision on the content of the final PROM	Clinician collaborators (2-3) Patient collaborator representatives (1-3)
Stage 4: preliminary measures of change			
	Online survey (time 2)	Gather secondary time point data for test-retest reliability and associated change analyses	Patients as research participants (>20)
	Cognitive interviews	Qualitative data to inform and help interpret minimum important difference using mixed methods	Patients as research participants (10)
	MAG 4	Discuss and review quantitative and qualitative data on MCID^g^. Reach consensus on recommendations	Clinician collaborators (2-3) Patient collaborators (1-3)

^a^ISBUS: Idiopathic Multicentric Castleman Disease Symptom Burden Scale.

^b^MAG: Multistakeholder Advisory Group.

^c^EAB: External Advisory Board.

^d^PROM: patient-reported outcome measure

^e^PAG: Patient Advisory Group.

^f^iMCD: idiopathic multicentric Castleman disease.

^g^MCID: minimal clinically important difference.

### Stage 1: Item Generation

As per FDA recommendations to leverage existing patient data wherever possible [30], existing evidence and expert opinion will be used to draft a long list of symptoms for the symptom burden scale [17]. As the literature on symptom burden in iMCD is scarce, a recent international survey of symptom burden in people living with iMCD is used as the primary empirical source [8], supplemented with evidence from a clinical trial in MCD using the MCD-SS [16]. These studies were conducted by coauthors of this protocol, who are experts in iMCD. Alongside this long list, draft PROM content (including instructions, possible response options, and recall period) will be generated by the research team with reference to best practice criteria in PROM development, including criteria developed by the coauthors based on their experience developing PROMs [31]. Draft PROM content will be reviewed and finalized at the first MAG meeting, with collaborative input from clinicians and patient representatives. This helps ensure initial content validity for the draft content formally assessed in stage 2 (ie, that the draft symptom list makes sense, contains symptoms potentially relevant to people with iMCD, and is comprehensive in scope). Triangulating concepts through multiple sources (ie, in this case, existing literature, clinical experts, and patient input) is a recommended approach in rare diseases [20]. In the context of an ultra-rare condition, reliance on existing data and multisource evidence generation at this stage is particularly important, as opportunities for extensive and representative primary data collection through qualitative interviews are limited by small and geographically dispersed patient populations. Organizations such as ISPOR (International Society for Pharmacoeconomics and Outcomes Research) explicitly recognize the need for flexibility and pragmatism when working in rare diseases [20], and adaptations to item generation are aligned with recommendations for rare disease outcome measure development [32].

### Stage 2: Item Testing and Refinement

#### Overview

Stage 2 of the project involves qualitative primary data collection via one-on-one online cognitive debriefing interviews. These interviews are designed to assess the content validity of the draft PROM generated in stage 1 and ensure that meaningful iMCD symptoms are identified and clearly described [17,33]. Content validity is an integral form of validity for a PROM and consists of comprehensibility (are each item and the PROM content understood as intended by the target population?), relevance (are the items and response options relevant to the construct of interest and target population?), and comprehensiveness (is anything of importance to the target population missing?) [34].

#### Participants and Recruitment

Participants in this study are required to have, at a minimum, a self-reported diagnosis of iMCD (diagnosis relayed to them by their physician) and are identified via two routes: (1) the Castleman’s Disease Collaborative Network (CDCN) and (2) clinicians (at clinical sites) who are treating patients with iMCD in the geographical regions of recruitment. The CDCN will facilitate recruitment by distributing a patient flyer throughout its network and is engaged proactively in the design, implementation, and dissemination of recruitment materials for ISBUS. Affiliated clinicians at clinical sites will also identify and distribute invitational flyers to patients they are treating for iMCD. Potential participants indicate their willingness to take part (and complete informed consent) by following the link on the flyer (and liaising with the researchers as necessary, eg, to schedule interviews for stage 2).

A multinational approach to recruitment is adopted due to the extremely rare nature of iMCD, with stage 2 recruitment available in affiliated English-speaking countries (ie, Australia, Canada, New Zealand, the United Kingdom, and the United States). From stage 3 onward, recruitment is extended to Brazil only for patients with a certain level of English proficiency (see below). Brazilian patients will not form part of the core group for analysis because of the potential for differences in understanding due to language considerations. To help ensure that people taking part in the study are genuine patients with iMCD, a screening filter has been developed with clinical experts that operationalizes the inclusion and exclusion criteria for this project (Textbox 1) [35]. This self-report filter is applied during the identification of new participants and the consent process for stages 2 and 3 (questions 2-7 in Multimedia Appendix 1 provide details of the eligibility filter used).

**Inclusion and exclusion criteria for patient involvement in the Idiopathic Multicentric Castleman Disease Symptom Burden Scale (ISBUS).**

**Inclusion criteria**

- Capacity to provide informed consent
- Self-reported physician-confirmed diagnosis of idiopathic multicentric Castleman disease (iMCD), including iMCD with thrombocytopenia, anasarca, fever, reticulin fibrosis and renal dysfunction, and organomegaly; iMCD with idiopathic plasmacytic lymphadenopathy; or iMCD not otherwise specified
- Adults (aged ≥18 years)
- Fluent in English

**Exclusion criteria**

- Lacking capacity to provide informed consent
- Self-reported positive test result for HIV or human herpesvirus 8, or diagnosis of or treatment for lymphoma, myeloma, or POEMS (polyneuropathy, organomegaly, endocrinopathy, monoclonal gammopathy, and skin changes)
- Children (aged <18 years)
- Not fluent in English

As iMCD is a rare condition, a realistic and practical approach is adopted to determine the target sample size, in accordance with similar PROM development projects. In the cognitive debriefing interviews, 10 people living with iMCD will be recruited to help validate and refine the content of the PROM. This is consistent with the sample size used in content validation as part of the development of a novel PROM by coauthors of this protocol in another rare condition (Duchenne muscular dystrophy [36]), an independent PROM developed in a rare disease (amyloid transthyretin amyloidosis [37]), and the Consensus-Based Standards for the Selection of Health Measurement Instruments (COSMIN) recommendation of a sample of at least 7 people [38]. It also aligns with the perspective that 6 to 12 interviews are often sufficient to identify the most important insights [20,39]. Given available resources and the challenges in recruiting fully representative samples in rare diseases, a pragmatic, convenience-based sampling method is adopted [30]. Variability in key disease characteristics (ie, length of illness and symptom severity) will be monitored and reported transparently. To maximize recruitment potential, patients who are members of the PAG are eligible to take part in the studies themselves, providing 2 levels of lived experience contribution.

#### Data Collection

Following best practice, a topic guide for the interviews will be used with questions and prompts for cognitive interviewing that follow guidance defined by ISPOR [40] (Multimedia Appendix 2). Prior to the interview, informed consent and selected background (clinical and sociodemographic) characteristics of the participants (including gender, age, ethnicity, employment status, income level, educational level, marital status, questions on disease severity, and questions on treatment) will be collected using an online survey. Interviews will be recorded and transcribed using intelligent verbatim transcription (being anonymized in the process). While ideally iterative, one round of cognitive interviewing is proposed in this study given available resources and recruitment challenges in ultra-rare diseases [32]. Instead, refinements made during cognitive interviewing are triangulated with patient and clinical input (PAG and MAG) to ensure lived experience input into decision-making. A similar approach has been adopted in the development of other rare disease PROMs [36,41].

The interviews are scheduled to last up to 1 hour, and the setting for the interviews is online. This choice of setting is both pragmatic, helping to enable efficiency in a rare disease [20], and methodologically sound. Research supports the use of online interviewing and the digital administration of PROMs as broadly equivalent to face-to-face or paper-based methods [42,43]. Participants receive an Amazon e-voucher of equivalent value in their own currency for taking part in the study.

#### Analysis

The transcribed qualitative data will be analyzed against an existing framework of themes for collating evidence on content validity in NVivo using framework analysis [44], a common approach to analyzing qualitative cognitive debriefing data [45,46]. Framework analysis is a systematic yet flexible approach unaligned with any specific epistemological, philosophical, or theoretical stance [47]. Reflexivity will be addressed by recognizing and transparently reporting the potential influence of researchers’ characteristics and a priori expectations on analytic decisions, alongside the use of team-based coding discussions to critically examine and moderate bias. The trustworthiness of the analysis will be addressed in several ways. Credibility will be enhanced through peer debriefing with patient representatives and researcher triangulation. Dependability will be strengthened through dual coding of the transcripts. Confirmability will be supported by an audit trail documenting analytic decisions and grounding findings in the qualitative data. Transferability will be enhanced through thick description of the research context, data, and findings [48].

Evidence from the framework analysis will be summarized qualitatively, and recommended revisions to the draft PROM will be tabulated for discussion with the PAG (session 1) and MAG (session 2). This may include revisions to items and PROM content, as well as the addition and removal of items. Any revisions and the rationale for them will be systematically tracked in an item-tracking matrix to provide rigor in the evidence base supporting PROM development [17,40]. Throughout item refinement and PROM design, the perceived respondent burden of ISBUS will be assessed through discussions with advisors in the PAG and MAG.

### Stage 3: Final Item Selection

#### Overview

Stage 3 of the project involves an online quantitative survey, in which the refined draft PROM (from stage 2) is administered alongside additional sociodemographic questions, clinical questions, and validated measures of symptom severity or HRQoL. The objective of this stage is twofold. First, to assess how the draft PROM performs statistically, with a view to making informed item selection decisions to reduce the content in the final PROM and maximize parsimony. Second, to provide preliminary psychometric evidence on the reliability and validity of the final PROM, including structural validity, hypothesis testing for construct validity, and internal consistency.

#### Participants and Recruitment

Participants for stage 3 are recruited via the same 2 pathways as described in stage 2 (ie, CDCN and clinician flyer with survey link), using convenience sampling to recruit as many eligible participants living with iMCD as possible. An ideal target sample size is ≥100, as simulations have suggested that samples of 100 and above produce more stable estimates in advanced psychometric analyses used in PROM development, such as Rasch model analysis [49]. To maximize the pool of potential recruits, patients who participated in stage 2 will be invited to participate in stage 3. Nevertheless, a sample of 100 may not be achievable given the rarity of the condition. Instead, an absolute minimum target sample size of 50 is adopted, as there is some evidence that this is a reasonable absolute minimum for conducting exploratory factor analysis [50]. Psychometric analyses will be adapted, as appropriate, to the final sample size (details provided in the Analysis subsection). Recruitment will continue until the minimum target sample is achieved.

As part of additional exploratory work for one of the project collaborators, English-speaking patients will be recruited from a health care site in Brazil. They are required to self-declare at least a B2 English language level [51]. These participants will be included in exploratory analyses only and not as part of the PROM development described in this protocol.

#### Data Collection

Survey content (Multimedia Appendix 1) will be agreed on alongside the PAG and clinical advisors prior to launch. The setting for the survey will be online to maximize recruitment numbers in a rare disease. Research suggests that electronic and paper-based PROM administration is broadly equivalent [43], and changing the mode of administration represents a minor to moderate modification that is unlikely to have a significant impact on scores [52]. However, it should be transparently acknowledged that mode of administration can affect who completes the survey (ie, the sample characteristics) [53,54].

After following a link to the online survey, participants provide informed consent and complete the iMCD screener described in stage 2. Those who pass the screener are considered eligible and proceed to answer sociodemographic and clinical background questions (ie, age, gender, country of residence, employment status, educational level, ethnicity, relationship status, age at the time of iMCD diagnosis, and iMCD treatment). Participants are then asked a single question on the impact of their iMCD symptoms on their daily activities (split into 3 options: no symptoms, symptoms but no impact on daily activities, and symptoms that are impacting daily activities).

Following these background questions, participants complete a draft version of the ISBUS PROM. Participants are then asked to rate the perceived importance of including each draft PROM symptom item in the final questionnaire on a 4-point Likert scale (0=not at all important; 3=very important). Participants then complete a 100-point visual analog scale with the stem, “Thinking about iMCD, how would you rate your quality of life during the past seven days?” (0=worst imaginable quality of life; 100=best imaginable quality of life). Next, participants are asked to complete the Patient Health Questionnaire-15 (PHQ-15), which measures physical symptoms [55], and the EQ-5D-5L (which measures health status [56]) for their country (US English was used for Brazil). Finally, participants are asked to leave their email address for reimbursement and to be contacted about participation in stage 4. Participants receive an Amazon e-voucher for taking part in the study (in their respective currency).

The 2 comparator measures were selected a priori based on their conceptual relevance and established psychometric performance. The PHQ-15 is a measure of somatic symptom severity that assesses the burden of common physical symptoms and has been widely validated across clinical populations. The EQ-5D-5L was selected to assess the relationship between symptom burden and broader HRQoL. We expect to observe at least moderate positive correlations between ISBUS and the PHQ-15 and moderate negative correlations between ISBUS and the EQ-5D-5L (ie, *r*_s_≥0.30). Only 2 additional PROMs were included to manage participant burden, given the length of the draft ISBUS PROM administered and the additional questions included.

#### Analysis

Table 2 outlines the planned psychometric analyses to be conducted as part of the ISBUS project. Analyses will be adapted to the actual sample size achieved (ie, ≥50 or ≥100) and will include, at the very least, exploratory factor analysis to assess structural validity; correlations and group comparisons for construct validity; and Cronbach α for internal consistency. Given the small sample size expected and the likely skewed distribution, it is anticipated that nonparametric statistics will be used (eg, Spearman ρ correlations and unweighted least squares estimation) [20,57].

**Table 2:** Planned psychometric analyses for the ISBUS^a^.

Stage and measurement property		Analysis	Evaluative criteria
Stage 3: final item selection			
	Structural validity		
		If n≥50 but <100: exploratory factor analysis using unweighted least squares for small samples	Loadings ≥0.40 Communalities ≥0.30 Chi-square test *P*>.5 RMSEA^b^<0.08 TLI^c^≥0.90 RMSR^d^≤0.08
		If n≥100: CFA^e^ or EFA^f^; Rasch modelling	CFA: same criteria as EFA Rasch: fit residuals between −2.5 and 2.5 Ordered thresholds
	Hypothesis testing for construct validity		
		Correlate ISBUS burden score with iMCD^g^ quality of life VAS^h^, PHQ-15^i^ score, and EQ-5D-5L utility	At least moderate correlations (rs≥0.30)
		Compare participants with symptoms impacting daily activities vs those without such impact	At least a moderate difference (d≥.05)
	Internal consistency		
		Cronbach α	Cronbach α≥0.70
Stage 4: preliminary measures of change			
	Test-retest reliability		
		ICC^j^ in participants reporting no change in health status	ICC≥0.70
	Measurement error		
		Standard error of measurement and MDC95^k^ in participants reporting no change in health status	MDC95<MIC^l^
	Responsiveness		
		SRM^m^ for improved, stable, and worsened group	SRM≥0.2 (improved and worsened group) and <0.2 (stable group)
		ROC AUC^n^ for improved subgroup	ROC AUC≥0.70 (improved group)
	Meaningful change (MIC)		
		Anchor-based MIC (mean and median improvement) for the minimally improved group	Correlation between anchor and change ≥0.30
		Distributional analyses (0.5 × SD, standard error of measurement) in the improved health group	MIC>MDC95
		Responder difference (mean and/or binary method)	More responders in the improved group than in the nonimproved group
		Triangulate quantitative and qualitative data and expert opinion to refine the MCID^o^ estimate	Used to inform the final MCID recommendation (no threshold applied)

^a^ISBUS: Idiopathic Multicentric Castleman Disease Symptom Burden Scale.

^b^RMSEA: root-mean-square error of approximation.

^c^TLI: Tucker-Lewis index.

^d^RMSR: root-mean-square residual.

^e^CFA: confirmatory factor analysis.

^f^EFA: exploratory factor analysis.

^g^iMCD: idiopathic multicentric Castleman disease.

^h^VAS: visual analog scale.

^i^PHQ-15: Patient Health Questionnaire-15.

^j^ICC: Intraclass correlation coefficient.

^k^MDC95: minimum detectable change at the 95% confidence level.

^l^MIC: minimum important change.

^m^SRM: standardized response mean.

^n^ROC AUC: receiver operating characteristic area under the curve.

^o^MCID: minimal clinically important difference.

### Stage 4: Preliminary Measures of Change

#### Overview

In order to provide initial insights into meaningful change scores on the new symptom burden scale, a mixed methods approach will be used. First, the draft PROM will be readministered to all participants from stage 3, alongside a global measure of perceived change in health status, after 2 months (informed by expert clinical opinion). This will allow estimates of stability (test-retest reliability and measurement error) in those without change, and estimates of change (responsiveness, minimal important change [MIC], otherwise known as meaningful score difference [58]) in those with change. Second, as iMCD is a rare disease, it is possible that insufficient participants are recruited at follow-up and/or insufficient change is observed to reliably estimate the MIC. Accordingly, in addition to quantitative estimates, semistructured qualitative debriefing interviews will be carried out with patients who have completed the survey at both time points to understand what constitutes a meaningful improvement from the patients’ perspective [19,20]. By triangulating this information with expert advisory input, the aim is to provide preliminary evidence on meaningful change using a natural history (ie, noninterventional) approach.

#### Participants and Recruitment

In stage 4, as many participants as possible who completed the stage 3 survey will be recruited to complete the survey a second time (by direct email invitation). A minimum target sample of at least 20 responses is established to help estimate test-retest reliability. However, the actual number of participants recruited may vary, and analyses will be adapted and interpreted with this in mind. Supplementary cognitive interviews to help support meaningful change estimates will be carried out with 10 people living with iMCD. These participants will be purposively sampled from those completing the stage 4 survey, aiming to ensure sufficient breadth in symptom burden (stage 4), perceived change in health (stage 3 to stage 4), and gender [20].

#### Data Collection

All stage 3 participants will receive an invitation with a link to the stage 4 online survey by email 2 months after they initially completed the survey. The stage 4 survey is a reduced form of the stage 3 survey, omitting most of the background questions (only questions on treatment remain) and the questions on the importance of symptoms. In addition, in order to measure and benchmark clinical change (or lack thereof) in participants’ perceived health, a self-report global impression of change will be used as the primary anchor [59]. The health transition item from the Short Form 36 Health Survey (ie, compared to when you last completed this questionnaire, would you say your overall health has improved a lot, improved somewhat, stayed the same, worsened somewhat, or worsened a lot) will be adapted to estimate changes in health status [60]. Global anchors capture the multidimensional experience of health and are widely used and understood. However, as a secondary construct-specific anchor, existing estimates of MIC for PHQ-15 and/or preestablished clinical cutoffs for the measure will be used [55,61].

Using a semistructured topic guide (Multimedia Appendix 3), the supplementary cognitive interviews are intended to identify meaningful changes from the patients’ perspective. Specifically, after consent procedures, participants will complete the shortened (finalized) version of the novel PROM to refamiliarize themselves with the instrument. They will then be shown their symptom profile (and overall burden score) from the stage 4 survey on screen. Participants will then be asked questions to identify what the minimum improvement in their symptom profile would be for them to consider meaningful. Finally, participants’ understanding of the global impression of change anchor (and what influenced their response) will be explored to help understand the quantitative results.

#### Analysis

As this study uses a natural history framework for exploring change (ie, it is noninterventional), all change statistics should be considered informative but preliminary only. Reliability and measurement error statistics can be calculated in the subsample of participants whose health status “stayed the same” between stage 3 and stage 4. Specifically, the intraclass correlation coefficient will be used to assess test-retest reliability, while the standard error of measurement and minimum detectable change at the 95% confidence level will be used to estimate measurement error in “stable” participants.

Preliminary analyses of responsiveness (ie, in the absence of an intervention) will be estimated using the standardized response mean (for improved, stable, and worsened participants) and the receiver operating characteristic area under the curve for the improved subgroup.

To estimate improvement-based MIC, the mean and median change in symptom burden (between stage 3 and stage 4) among participants who reported a minimal improvement in health (ie, “improved somewhat”) will form the primary anchor-based MIC. A secondary anchor approach will compare change in symptom burden with change in PHQ-15 scores, with an “improved” group defined by (1) a PHQ-15 score greater than or equal to the estimated MIC for the PHQ-15 of 3 points [61] and/or (2) categorical improvement in PHQ-15 clinical cutoff groups (eg, from “moderate” to “mild” symptoms) [55]. These anchor methods will be supplemented with distribution-based analyses using standard approaches (ie, 0.5 × SD and standard error of measurement) [59]. Change in worsened groups will be calculated and reported for comparative purposes but is not the primary focus of interest. Finally, responder difference will be estimated using (1) the binary method, whereby the proportion of people in improved vs not improved subgroups experiencing improvement above the MIC is estimated; and (2) the mean method, whereby the difference in mean burden change for the improved vs not improved subgroups is calculated [62].

Analysis of the qualitative interviews will involve a quantitative estimate of meaningful improvement and thematic coding of supportive quotes to understand patients’ rationale for meaningful change [63]. Qualitative data will be analyzed using a hybrid inductive-deductive content analysis approach [64], commonly used in interviews with concept elicitation elements [65,66]. Reflexivity and trustworthiness will be addressed using the same methods as in stage 2. Ultimately, recommendations for a preliminary minimal clinically important difference (improvement-focused) will be informed by input from clinical and patient advisors in the MAG, triangulating clinical opinion with the available quantitative and qualitative data on meaningful change. Values will be rounded to possible scores on the instrument where relevant, and it is possible that a range (eg, with a lower and upper bound) rather than a single value may be proposed [58].

### Ethical Considerations

The ISBUS project will be conducted in line with the ethical principles underlying the Declaration of Helsinki and best practices in research. The study received ethical approval in each country involved in the project. Specifically, in the United Kingdom, the project received National Health Service Research Ethics Committee and Health Research Authority approval on December 20, 2023 (23/WS/0160); Advarra US/Canada initial approval on March 14, 2024 (Pro00077570), and multisite approval on June 10, 2025 (MOD02596629); Bellberry Australia approval on April 17, 2024 (2024-02-170); South Eastern Sydney Local Health District Human Research Ethics Committee (Regis) approval on November 12, 2025 (2024/ETH01650); Health and Disability Ethics Committees New Zealand approval on September 22, 2025 (2025 EXP 22447); and Plataforma Brasil approval on December 11, 2025 (85853725.5.0000.5483).

Informed consent will be obtained from all participants in all stages of the project involving primary research, including reconsent for the follow-up survey in stage 4. At the time of consent, personal identifying data will be collected for this study to facilitate reimbursement, enable future communication with participants consenting to be recontacted for stages 3 and 4, and comply with pharmacovigilance reporting. These personal identifying data will be stored separately from all other study data and only for as long as needed. Appropriate measures are in place to safeguard participant information in a dedicated data management plan, which has been approved by the ethics review boards detailed above. Anonymized research data will be used to support study outputs, and no identifiable information will be published.

Participants will be reimbursed for their time with an Amazon voucher of 50 units in their local currency (eg, US $50) for survey participation (stages 3 and 4) and 75 units in their local currency (eg, US $75) for interview participation (stages 2 and 4). The exception is Brazil, where reimbursement for research participation is not permitted.

## Results

The ISBUS project was funded in June 2023 and is ongoing at the time of submission. Stage 1 (item generation) began at the start of the project and concluded in December 2023. Stage 2 (item testing and refinement) started in January 2024; interviews with people living with iMCD (n=10) were conducted between May 2024 and September 2024, and this stage concluded in November 2024. Stage 3 began in December 2024. The first survey participant was enrolled in February 2025, and recruitment continued until November 2025, with 51 people living with iMCD completing the survey. Stage 3 concluded in January 2026, when the symptom burden scale was finalized. However, recruitment in Brazil (which is being used for comparative purposes only) is ongoing. Stage 4 is ongoing. As of March 2026, when the paper was submitted, 32 people had completed a follow-up survey, and the qualitative interviews on meaningful change were ongoing. A scale development paper (featuring the results from stages 1-3) is expected to be published in 2026, with a further paper on understanding change (stage 4) published in 2027. In accordance with established best practice [17], a user manual will be produced for the new PROM describing, among other aspects, how to score and administer the measure.

## Discussion

### Summary

The ISBUS project addresses a clear research gap and practical need for a condition-specific iMCD symptom burden scale developed using a patient-centered methodology, which is designed to capture what matters most to patients and help meet regulatory requirements for patient-centered evidence in health outcomes evaluation. The project has been designed in light of FDA recommendations for scale development [17], with pragmatic and practical considerations for available resources and working in an ultra-rare disease [19,20,30]. The work conducted in ISBUS will build upon a prior international iMCD symptom burden survey conducted by protocol coauthors, and the findings can be compared directly with this to explore areas of replicability [8,67]. Nevertheless, novel insights from sequential mixed methods research, including qualitative interviews and psychometrics on symptom burden, will contribute meaningful evidence to the literature on iMCD.

The novel PROM that arises from the ISBUS project will be supported by available evidence on the initial psychometric and measurement properties of the instrument. This is supported by a transparent audit trail of all decisions made during the development process. As with all PROM development work, the process is fundamentally iterative, and further studies will be encouraged to fully assess and amass a wider evidence base on the psychometric properties of the final PROM. While the primary intention of the new measure is to assess change in symptom burden in research and clinical trials, it is hoped that the measure will also have utility in routine clinical practice to benchmark and explore change for individual patients with iMCD. This will require work on implementation strategies, as well as further studies evaluating the ISBUS in clinical settings. The novel PROM will be designed with parsimony and minimizing patient burden in mind.

### Strengths and Limitations

A strength of the ISBUS approach is its focus on patient-centered methods, including the involvement of people with lived experience of iMCD throughout the project and as collaborators in decision-making. The study, wherever possible, follows best practices in PROM development [17] and triangulates multiple sources of evidence to derive a fit-for-purpose measure. A robust governance structure and the involvement of leading external advisors help to further enhance the rigor of the ISBUS project.

Several limitations should be noted. First, iMCD is a rare disease (with a prevalence of less than 7 cases per 1 million) [5], and pragmatic yet justified deviations from best practice have been made in this context, in consideration of available resources. The use of convenience sampling in stages 2 and 3, for example, means that the sample may not be fully representative of the broader iMCD cohort [30]. This concern is mitigated by the engagement of patient and clinician advisory groups to help interpret the results and inform decision-making through triangulated perspectives, as well as by transparently recording and reporting sample characteristics. The lack of direct concept elicitation interviews with patients in stage 1 is a limitation. While a multisource approach to item generation was used—including existing patient surveys, expert input, and input from patient representatives—we cannot fully rule out the possibility that some concepts may emerge only through dedicated concept elicitation interviews and therefore may not be comprehensively captured in the current approach.

Smaller samples are inevitable in rare disease research, and this limits what can be done in terms of psychometric analysis. The multinational approach of the ISBUS project helps to address this, but implicit in this approach is the assumption that the survey operates equivalently across different primarily English-speaking countries (due to the expected small sample sizes from different countries, it may be impractical to test this assumption formally). While samples could be expanded by recruiting patients with sufficiently analogous conditions [20], this decision risks undermining the goal of the novel PROM and its unique applicability to people living with iMCD. Instead, methods will be adapted for smaller samples, with any limitations transparently noted during dissemination [30]. In addition to the smaller sample size being challenging, the fact that iMCD is an episodic condition in which symptom burden is related to the activity of the disease and the treatment that the patient is receiving means that the timing of survey administration is important. We aim to address this by enrolling as many patients as possible in the hope that patients experiencing initial flares, relapses, and remissions will all be captured.

Another limitation to consider is the reliance on self-reported diagnosis. However, methods including a robust, clinically informed screener and dual recruitment routes (ie, via the CDCN and clinically verified patients with iMCD) help to offset this. Further, the reliance on online data collection is a pragmatic and advisable choice in rare disease research [19], but may limit inclusion of those who choose not to, or are unable to, take part in online studies. Reassuringly, research suggests switching from online to paper-based administration (or vice versa) is likely to have only a minor influence on responses [52]. Finally, the estimates of change in stage 4 should be considered preliminary only, as they are derived from a natural history approach (ie, noninterventional). While our approach is strengthened by triangulation of multiple methods, estimates such as the standardized response mean (responsiveness) may form a lower bound of sensitivity to change (as no direct study intervention was administered) and should be considered conservative.

### Conclusion

In summary, the ISBUS project will produce a novel PROM codeveloped with patients with iMCD to measure what matters to patients with iMCD. The novel measure will aim to achieve comprehensive coverage of important iMCD symptoms while minimizing respondent burden and achieving psychometric parsimony. It is anticipated that the new PROM will be used in iMCD research, as a secondary end point in trials, and as an exploratory tool in clinical settings.

## Data Availability

The datasets generated or analyzed during this study are not publicly available due to the sensitive nature of the data and the potential risk of participant identification in a rare disease population. Deidentified data may be available for research purposes upon reasonable request, subject to applicable governance requirements, participant consent, ethics approvals, and institutional and country-specific regulations. Requests for access to the data should be directed to Recordati Rare Diseases.

## Appendix

Multimedia Appendix 1 Survey outline for stage 3 (UK version).

Multimedia Appendix 2 Interview topic guide for stage 2.

Multimedia Appendix 3 Interview topic guide for stage 4.

## Abbreviations

CDCN: Castleman’s Disease Collaborative Network
COSMIN: Consensus-Based Standards for the Selection of Health Measurement Instruments
FDA: Food and Drug Administration
HHV-8: human herpesvirus 8
HRQoL: health-related quality of life
iMCD: idiopathic multicentric Castleman disease
ISBUS: Idiopathic Multicentric Castleman Disease Symptom Burden Scale
ISPOR: International Society for Pharmacoeconomics and Outcomes Research
MAG: Multistakeholder Advisory Group
MCD: multicentric Castleman disease
MCD-SS: MCD-Symptom Scale
MIC: minimal important change
PAG: Patient Advisory Group
PHQ-15: Patient Health Questionnaire-15
POEMS: iMCD associated with polyneuropathy, organomegaly, endocrinopathy, monoclonal plasma cell disorder, and skin changes
PROM: patient-reported outcome measure
