# Supplementary material for: Development and Validation of the Idiopathic Multicentric Castleman Disease Symptom Burden Scale (ISBUS): Protocol for an International Multistage Mixed Methods Study
Source: JMIR Res Protoc. 2026 Aug 11;15:e96022. doi: 10.2196/96022 (PMC13507722; doi:10.2196/96022)
Supplement: Supplementary file 1 [file resprot_v15i1e96022_app1.docx]

**Appendices**

**Appendix 1 – Survey outline for Stage 3 (UK version)**

**Producing a novel iMCD Symptom Burden Scale (ISBUS)**

**Draft survey (Stage 3)**

[Insert Study Information Sheet and Participant Consent Form]

**SCREENING QUESTIONS AND SOCIODEMOGRAPHIC INFORMATION**

Thank you for completing the consent form. We would like to ask you to fill out some background questions to help us assess your eligibility to participate in our study. Please complete these to the best of your knowledge and ability.

1. Where did you hear about this study?

|  | The Castleman Disease Collaborative Network |
| --- | --- |
|  | My iMCD clinician |

[If 1. Where did you hear about this study? – ‘My iMCD clinician’ is selected]

1a. At which NHS site do you receive treatment for iMCD?

|  | Oxford University Hospitals NHS Trust |
| --- | --- |
|  | University College London Hospitals NHS Foundation Trust |
|  | Other (please specify)  --------------------------------------- |

2. Has your physician told you that you have Castleman Disease?

|  | Yes |
| --- | --- |
|  | No [Exclude from survey – ineligible] |
|  | I cannot recall |

3. Which type of Castleman Disease has your physician diagnosed you with?

|  | Unicentric Castleman Disease [Exclude from survey – ineligible] |
| --- | --- |
|  | Multicentric Castleman Disease |
|  | I cannot recall |

4. Has your physician told you that you have idiopathic Multicentric Castleman’s Disease (iMCD)

|  | Yes |
| --- | --- |
|  | No [Exclude from survey – ineligible] |
|  | I cannot recall [Exclude from survey IF participant also answered ‘I cannot recall’ to 3. Has your physician told you that you have Castleman Disease? AND 4. Which type of Castleman Disease has your physician diagnosed you with?] |

5. Has your physician told you that you are HIV positive?

|  | Yes [Exclude from survey – ineligible] |
| --- | --- |
|  | No |
|  | I cannot recall |

6. Has your physician told you that you are HHV-8 positive?

|  | Yes [Exclude from survey – ineligible] |
| --- | --- |
|  | No |
|  | I cannot recall |

7. Has your physician diagnosed and/or treated you for lymphoma, myeloma or POEMS (Polyneuropathy, organomegaly, endocrinopathy, monoclonal gammopathy, and skin changes)?

|  | Yes [Exclude from survey – ineligible] |
| --- | --- |
|  | No |
|  | I cannot recall |

8. Have you had one or more biopsies of lymph nodes/tissues in different parts of your body or other surgery to diagnose your (iMCD)?

|  | Yes |
| --- | --- |
|  | No |
|  | I cannot recall |

9. Are you registered with the ACCELERATE registry?

|  | Yes |
| --- | --- |
|  | No |
|  | I cannot recall |

10. What is your age (in years)?

-----------------------------------------------------------------

11. What is your gender?

|  | Woman |
| --- | --- |
|  | Man |
|  | I prefer to identify my gender in another way |
|  | Prefer not to say |

12. What is your country of residence?

|  | Australia |
| --- | --- |
|  | Brazil |
|  | Canada |
|  | New Zealand |
|  | UK |
|  | US |
|  | Other (please specify)  ----------------------------------------------------------------- |

13. Which best describes your primary employment status?

|  | Employed (including full-time, part-time, self-employed, voluntary or on a temporary leave of absence, such as parental or sickness leave) |
| --- | --- |
|  | Unemployed, seeking work |
|  | Unemployed, due to disability or sickness |
|  | Student/training |
|  | Retired |
|  | Caring for family |
|  | Looking after the home |
|  | Other (please specify)  ----------------------------------------------------------------- |
|  | Prefer not to say |

14. Did your education continue after the minimum school leaving age?

|  | Yes |
| --- | --- |
|  | No |
|  | Prefer not to say |

[If 14. ‘Did your education continue after the minimum school leaving age?’ – ‘Yes’ is selected]

14a. Do you have a degree or equivalent professional qualification?

|  | Yes |
| --- | --- |
|  | No |
|  | Prefer not to say |

15. Which based describes your ethnicity?

|  | Asian or Asian British |
| --- | --- |
|  | Black, Black British, Caribbean or African |
|  | Mixed or multiple ethnic groups |
|  | White |
|  | Other ethnic group |
|  | Prefer not to say |

16. What best describes your relationship status?

|  | Single |
| --- | --- |
|  | Married or in a civil partnership |
|  | Living with partner/cohabiting |
|  | Divorced |
|  | Widowed |
|  | Prefer not to say |

17. What was your age at diagnosis of iMCD (in years)?

-----------------------------------------------------------------

18. Are you currently receiving/have you ever received treatment for iMCD?

|  | Yes |
| --- | --- |
|  | No |
|  | I do not recall |

[If 18. ‘Are you currently receiving/have you ever received treatment for iMCD?’ – ‘Yes’ is selected]

18a. Please indicate whether you have ever had or you are currently on any of the treatments below for iMCD (please select all that apply). For any treatments that you have received please provide your most recent frequency of treatment (for example in days or weeks).

|  | Have you had this treatment? | | How often did you/do you receive this treatment? |
| --- | --- | --- | --- |
|  | I have been on this treatment | I am still on this treatment | I receive/received this treatment every… (please specify time frame, e.g. days, weeks) |
| Siltuximab (Sylvant) | □ | □ |  |
| Rituximab (Rituxan, Riabni, Riximyo, Ruxience, Truxima, others) | □ | □ |  |
| Tocilizumab (Actemra, others) | □ | □ |  |
| Ruxolitinib (Jakafi) | □ | □ |  |
| Lenalidomide (Revlimid, others) | □ | □ |  |
| Thalidomide (Thalomid, others) | □ | □ |  |
| Cyclosporine (Sandimmune, Neoral, Gengraf) | □ | □ |  |
| Anakinra (Kineret) | □ | □ |  |
| Steroids/corticosteroids (e.g., prednisone (Rayos, Prednisone Intensol, Deltasone), dexamethasone (Ozurdex, Maxidex, DexPak, LoCort, ZonaCort) etc.) | □ | □ |  |
| Everolimus (Zortress, Afinitor Disperz, Afinitor) | □ | □ |  |
| Sirolimus (Repamune) | □ | □ |  |
| Chemotherapy (other than any of the above) | □ | □ |  |
| Other, please name: ___________________ | □ | □ |  |

19. Which of the following applies to your current experience living with iMCD?

|  | The symptoms I am experiencing are having an impact on my daily activities, such as my ability to do housework, socialising, working etc. |
| --- | --- |
|  | The symptoms I am experiencing are not having an impact on my daily activities, such as my ability to do housework, socialising, working etc. |
|  | I am not currently experiencing any symptoms. |

**iMCD Symptom Burden Scale**

This questionnaire is asking about symptoms of idiopathic multicentric Castleman disease (iMCD) and how they have impacted your life during the **past seven days**.

For each symptom below, we would like you to consider how often it applied to you during the **past seven days**, and, for the symptoms you experienced, we would like you to consider how much of an impact it had on your life during the **past seven days**.

**SYMPTOM FREQUENCY**

On this page, please read each symptom below and think about how often it applied to you during the past seven days. Please select the answer that best applies to you.

Due to iMCD, during the past seven days I experienced...

|  | None of the time | Sometimes | Often | Most or all of the time |
| --- | --- | --- | --- | --- |
| Tiredness (i.e., either physical and/or mental tiredness) |  |  |  |  |
| Exhaustion |  |  |  |  |
| Low energy |  |  |  |  |
| Physical weakness |  |  |  |  |
| Dizziness (including feeling faint, weak, or unsteady) |  |  |  |  |
| Difficulty concentrating |  |  |  |  |
| Trouble thinking clearly |  |  |  |  |
| Memory problems |  |  |  |  |
| Depression |  |  |  |  |
| Sadness |  |  |  |  |
| Mood swings |  |  |  |  |
| Anxiety |  |  |  |  |
| Loss of libido (sexual desire) |  |  |  |  |
| Daytime sweating (not related to heat or exercise) |  |  |  |  |
| Nighttime sweating (not related to heat or exercise) |  |  |  |  |
| Fever |  |  |  |  |
| Unpredictable changes in temperature |  |  |  |  |
| Shortness of breath |  |  |  |  |
| Numbness/tingling (in my hands and feet) |  |  |  |  |
| Itchiness |  |  |  |  |
| Fluid or swelling in part(s) of my body |  |  |  |  |
| Lump(s) in part(s) of my body |  |  |  |  |
| Muscle cramps |  |  |  |  |
| Pain in my abdomen |  |  |  |  |
| Headaches |  |  |  |  |
| Unintentional weight gain |  |  |  |  |
| Unintentional weight loss |  |  |  |  |
| Lack of appetite |  |  |  |  |
| Nausea |  |  |  |  |
| Dry mouth |  |  |  |  |

| Page Break |  |
| --- | --- |

**SYMPTOM IMPACT**

[Participants will only see symptoms below that they indicated having experienced more than ‘None of the time’ in the frequency questions above]

On this page, please read each symptom below and think about how much of an impact it had on your life during the **past seven days**. Please select the answer that best applies to you.

This iMCD symptom had the following impact on my life during the **past seven days**...

|  | No impact | Slight impact | Moderate impact | Severe impact |
| --- | --- | --- | --- | --- |
| Tiredness (i.e., either physical and/or mental tiredness) |  |  |  |  |
| Exhaustion |  |  |  |  |
| Low energy |  |  |  |  |
| Physical weakness |  |  |  |  |
| Dizziness (including feeling faint, weak, or unsteady) |  |  |  |  |
| Difficulty concentrating |  |  |  |  |
| Trouble thinking clearly |  |  |  |  |
| Memory problems |  |  |  |  |
| Depression |  |  |  |  |
| Sadness |  |  |  |  |
| Mood swings |  |  |  |  |
| Anxiety |  |  |  |  |
| Loss of libido (sexual desire) |  |  |  |  |
| Daytime sweating (not related to heat or exercise) |  |  |  |  |
| Nighttime sweating (not related to heat or exercise) |  |  |  |  |
| Fever |  |  |  |  |
| Unpredictable changes in temperature |  |  |  |  |
| Shortness of breath |  |  |  |  |
| Numbness/tingling (in my hands and feet) |  |  |  |  |
| Itchiness |  |  |  |  |
| Fluid or swelling in part(s) of my body |  |  |  |  |
| Lump(s) in part(s) of my body |  |  |  |  |
| Muscle cramps |  |  |  |  |
| Pain in my abdomen |  |  |  |  |
| Headaches |  |  |  |  |
| Unintentional weight gain |  |  |  |  |
| Unintentional weight loss |  |  |  |  |
| Lack of appetite |  |  |  |  |
| Nausea |  |  |  |  |
| Dry mouth |  |  |  |  |

**IMPORTANCE OF SYMPTOMS**

On this page, please read each symptom below and think about **how important you think it is to include in the questionnaire assessing symptom burden of iMCD**.

We want to know this information because we will need to make decisions to reduce the number of questions within the final symptom burden questionnaire.

To include in the final questionnaire, I think this iMCD symptom is…

|  | Not at all important | Slightly important | Moderately  important | Very  important |
| --- | --- | --- | --- | --- |
| Tiredness (i.e., either physical and/or mental tiredness) |  |  |  |  |
| Exhaustion |  |  |  |  |
| Low energy |  |  |  |  |
| Physical weakness |  |  |  |  |
| Dizziness (including feeling faint, weak, or unsteady) |  |  |  |  |
| Difficulty concentrating |  |  |  |  |
| Trouble thinking clearly |  |  |  |  |
| Memory problems |  |  |  |  |
| Depression |  |  |  |  |
| Sadness |  |  |  |  |
| Mood swings |  |  |  |  |
| Anxiety |  |  |  |  |
| Loss of libido (sexual desire) |  |  |  |  |
| Daytime sweating (not related to heat or exercise) |  |  |  |  |
| Nighttime sweating (not related to heat or exercise) |  |  |  |  |
| Fever |  |  |  |  |
| Unpredictable changes in temperature |  |  |  |  |
| Shortness of breath |  |  |  |  |
| Numbness/tingling (in my hands and feet) |  |  |  |  |
| Itchiness |  |  |  |  |
| Fluid or swelling in part(s) of my body |  |  |  |  |
| Lump(s) in part(s) of my body |  |  |  |  |
| Muscle cramps |  |  |  |  |
| Pain in my abdomen |  |  |  |  |
| Headaches |  |  |  |  |
| Unintentional weight gain |  |  |  |  |
| Unintentional weight loss |  |  |  |  |
| Lack of appetite |  |  |  |  |
| Nausea |  |  |  |  |
| Dry mouth |  |  |  |  |

Thinking about iMCD, how would you rate your quality of life during the past seven days?

(0 = worst imaginable quality of life, 100 = best imaginable quality of life).

Please give a number from 0 to 100

| 0 | 10 | 20 | 30 | 40 | 50 | 60 | 70 | 80 | 90 | 100 |
| --- | --- | --- | --- | --- | --- | --- | --- | --- | --- | --- |

[Insert EQ-5D-5L here]

[Insert PHQ-15 here]

Thank you for completing this survey. Please provide your email address, so you can be compensated for your time. We will contact you again about potential participation in a follow-up survey in 2 months’ time, but it is your choice whether to take part at that point.

Email address: ______________________________________

SUBMIT

**Differences for the 2-month follow-up survey**

The 2-month follow-up survey will have identical content to above, but with some minor differences. Sociodemographic questions 1-17 will not be repeated, but questions on treatment (18 and 19) will be asked again to check for any differences.

The following additional question will be added to the survey to provide a benchmark of clinical change (or lack thereof) in participants’ symptom burden:

“Compared to when you last completed this questionnaire, would you say your overall health has:

|  | Improve a lot |
| --- | --- |
|  | Improved somewhat |
|  | Stayed the same |
|  | Worsened somewhat |
|  | Worsened a lot” |

The survey will close with the following statement:

“Thank you for completing this survey. Please provide your email address, so you can be compensated for your time. We will contact you a final time about potential participation in a follow-up interview, but it is your choice whether to take part at that point.

Email address: ______________________________________”
