# Supplementary material for: Development and Validation of the Idiopathic Multicentric Castleman Disease Symptom Burden Scale (ISBUS): Protocol for an International Multistage Mixed Methods Study
Source: JMIR Res Protoc. 2026 Aug 11;15:e96022. doi: 10.2196/96022 (PMC13507722; doi:10.2196/96022)
Supplement: Supplementary file 2 [file resprot_v15i1e96022_app2.docx]

**Appendix 2 – Interview topic guide for Stage 2**

**Topic guide for Stage 2 – Cognitive interviewing**

*This is a semi-structured topic guide designed to guide the interviewer. It is not intended that all the questions listed below will be asked in each interview, or that they will be asked in the same order. It is designed to ensure all important content is covered across the interviews as a whole. The interviewer will be responsive to the participant and what they have to say about the questionnaire.*

1. **Introduction and consent**

[Formal online consent will be obtained prior to the interview]

- The interviewer will thank the participant for agreeing to take part in the interview.
- The interviewer will introduce themselves and the topic of discussion today.
- The interviewer will remind participants that they have given consent and check that they still wish to take part in the interview.
- Participants will be reminded of the salient aspects of informed consent, including the voluntary nature of their participation, right to withdraw, and confidential nature of the discussion/data.
- Participants will be reminded that no questions are compulsory if there is anything they do not want to discuss then that is up to them and we can simply move on.
- Participants will be reminded that the interview will take up to an hour to complete, but that they can stop at any time, or take a break, if they wish to do so.
- Participants will be reminded that the interview will be audio-recorded with their consent.
- The interviewer will ask for any questions and check that the participant is happy to continue before the recording is started.

[The interview recording will be started at this point]

2. **Interview guide**

**2.1.	Instructions**

[The participant will be directed to the instructions at the top of the questionnaire and asked to read them through if they have not already]

- Can you tell me, in your own words, what these instructions are asking you to do?
- Can you describe any confusion or difficulty you had in understanding these instructions?
- Are there any words or phrases that you would change to improve the instructions?

**2.2.	Recall period**

[The participant will be directed to the ‘past week’ recall period in bold in the instructions]

- Considering the symptoms listed in the questionnaire, are these symptoms that you might experience over a week?
- Can you describe your experiences of iMCD symptoms over the past week?
- Do you think a 1-week recall period is appropriate for this questionnaire? Would a shorter or longer record period be preferred? Why or why not?

**2.3.	Item content**

[Participants will be directed to and asked about the symptom questions in the questionnaire]

- What does [question content] mean to you?
- In your own words, could you explain what this question means?
- Is [question content] relevant to your experience of iMCD? Why or why not?
- For [selection of question content], which question do you prefer? Why?

**2.4.	Response options**

[Participants will be directed to the response choices in the questionnaire, including those related to frequency and related to impact]

- For the questions about frequency, please read the instruction and each of the choices and describe, in your own words, what they mean to you.
- For the questions about impact, please read the instruction and each of the choices and describe, in your own words, what they mean to you.
- Can you tell the difference between the different response choices? For example, ‘sometimes’ and ‘often’, or ‘slight impact’ and ‘moderate impact’? Why or why not?
- Do you think a 4-point scale is appropriate for reporting the frequency of these symptoms? Why or why not?
- Do you think a 4-point scale is appropriate for reporting the impact of these symptoms? Why or why not?
- Would you ever choose ‘Most or all of the time’? Why or why not?
- Can you describe an experience where you would choose ‘Severe impact’?

**2.5.	Comprehensiveness**

- Are there any other symptoms that you experience related to iMCD that are not covered in this questionnaire?
- Looking at whole questionnaire, are any of the questions redundant or unnecessary?

**2.6.	Format**

- Would you prefer to answer questions on the impact of each symptom directly after answering the question on its frequency, or would you prefer to answer all frequency questions first and then all the impact questions after?

3. **Close**

The interviewer will turn off the recording and ask the participant if they have any questions about the project. They will then thank the participant for their time.
