# Supplementary material for: Development and Validation of the Idiopathic Multicentric Castleman Disease Symptom Burden Scale (ISBUS): Protocol for an International Multistage Mixed Methods Study
Source: JMIR Res Protoc. 2026 Aug 11;15:e96022. doi: 10.2196/96022 (PMC13507722; doi:10.2196/96022)
Supplement: Supplementary file 3 [file resprot_v15i1e96022_app3.docx]

**Appendix 3 – Interview topic guide for Stage 4**

1. **Introduction, grounding and consent**

[Formal online consent will be obtained prior to the interview]

- The interviewer will thank the participant for agreeing to take part in the interview.
- The interviewer will introduce themselves and the topic of discussion today which is understanding what kinds of changes in your symptoms reported on the iMCD symptom burden questionnaire would feel meaningful to you personally.
- The interviewer will remind the participant that the interviewer would be looking at Global Burden Score at follow-up and the Individual Symptom Burden scores.
- The interviewer will inform the participant that they will ask questions to understand participant’s views around what constitutes the smallest meaningful difference to them in the scores of the questionnaire and individual question responses over time.
- The interviewer will remind participants that they have given consent and check that they still wish to take part in the interview.
- Participants will be reminded of the salient aspects of informed consent, including the voluntary nature of their participation, right to withdraw, and confidential nature of the discussion/data.
- Participants will be reminded that no questions are compulsory if there is anything they do not want to discuss then that is up to them, and we can simply move on.
- Participants will be reminded that the interview will take up to an hour to complete, but that they can stop at any time, or take a break, if they wish to do so.
- Participants will be reminded that the interview will be audio-recorded with their consent.
- The interviewer will ask for any questions and check that the participant is happy to continue before the recording is started.

[The interview recording will be started at this point].

2.  **Brief Re-Introduction to the Questionnaire**

- The interviewer will share their screen with the participant
- Present questionnaire and ask the participant to complete [on Qualtrics] the 12-item version of the ISBUS measure to refresh their memory about its content, as well as get a sense of the symptoms and burden they are currently experiencing any iMCD symptoms

3. **Explanation of the scores**

- The participant an explanation of how the frequency, impact and overall burden scores are calculated.
- Some illustrated examples are shown to the participant as well as all the possible combinations of scores for an individual symptom and how they are calculated.
- The interviewer will check participant understanding at this point and answer any questions they may have

4. **Discussion of 12-item Burden Scores**

- The participant is shown the 12-item scale with their individual burden score per symptom emphasising that this may not be representative of their symptom now.
- The domain / symptom-level meaningful change is elicited using the following steps:
  i. the participant is shown the score at item and total level [out of 36) when they completed T2 questionnaire
  ii. the participant is asked to use that burden profile as a starting point to help identify what the smallest amount of improvement in symptom burden would be classed as meaningful to them.
  iii. the participant is asked to think about the smallest meaningful improvement across their set of symptoms and identify which of these symptoms they would want to see improvement in. It might be just one or several symptoms that they think would make a difference for them.
  iv. Once a symptom is specified, the interviewer electronically moves symptom(s) with participant’s response and clarify if that is correct. In the instances where more than one symptom is identified, this process is iterated for each symptom. [if participant does not specify a change, the interviewer will use some prompts and examples to help them]
  v. The interviewer rephrases the answer(s) provided by the participant, asks a counter question and finally request the participant to confirm if they are happy with the choice(s).
  vi. The interviewer asks the participant to talk them through and explain to the interviewer why they have made the choices that they have
  vii. The interviewer notes down the changes, reflects these changes for the individual symptom and manually modifies the score and moves the burden slider on the screen to reflect the new score.
  viii. The participant is then shown the changes in symptoms and overall score and is asked whether they are satisfied that this represents the smallest change in iMCD symptom burden that would be meaningful for you
  ix. The participant will be given the chance to revisit their answer if at that point they do not think the minimum meaningful change is not accurate.

5. **Anchoring**

- The participant is reminded that as part of the second survey they completed in [interviewer to specify date], they were asked this question about whether your health had improved, worsened, or stayed the same since you first completed the questionnaire.
- The interviewer will ask them to rate their overall health today compared to the last time they completed this questionnaire.
- The interviewer will also probe the participant to find out what the latter took into consideration when completing this question.

6. **Close**

The interviewer will turn off the recording and ask the participant if they have any questions about the project. They will then thank the participant for their time and inform them about their voucher.
